# Remarks, Gleanings, and Extracts

**Published:** 1874-08

**Authors:** R. C. Word

**Affiliations:** Georgia


### Remarks, Gleanings and Extracts.

BY R. C. WORD, M.D., GEORGIA.

*Gratuities.*—Courtesy, and the generous spirit that should exist between medical men, would seem to require that one physician ought not to charge another for medical services to himself or family, and such is, and has been, the custom from time immemmorial. While this is the rule, it is tacitly understood to apply only to practitioners in the same community, where there is opportunity for reciprocity. It applies also to a visiting brother or his family who may chance to sojourn in the field occupied by another physician. Yet, in this case, if the services be long or protracted, the visiting brother should not accept the gratuity, but make compensation in cash, or by a present, equivalent in value to the service rendered.

Ex-doctors, or those who have retired from the practice and engaged in other pursuits, are not entitled to, and ought not to expect gratuitous services.

The idea, which seems to have established itself in the public mind, that physicians ought not to charge clergymen, has no just grounds to sustain it. Physicians, as a class, are benevolent and kind, and so often bestow their services gratuitously to ministers, that the community have come almost to the point of demanding it, and the minister to feel it his due. But where is the justice of requiring the physician to make this gratuity any more than the merchant, the grocer, or the butcher. Indeed, there is far less reason why the physician should do so, for it is well known that medical men, in their attention to the indigent sick, do an amount of gratuitous labor far surpassing that of all other callings combined.

*Therapeutic Review.*—The *Ravista Clinica de Bologna* gives occasionally an admirable summary of therapeutics, from which we borrow some paragraphs.

*Carbolic Acid* has been praised in prurigo and pruritus, subcutaneously injected in doses of about one centigramme of the acid mingled with water. It has been used externally in acute articular rheumatism as a liniment mingled with linseed oil.

*Arsenic* has been recently recommended in cases of strumous enlarged glands of the neck, and also in pellagra.

*Bromine.*—Inhalations of bromine have been used in croup and diphtheritis; 30 centigrammes of bromine, 30 of bromide of potassium, and 150 grammes of water are combined in a lotion; and a sponge imbibed with this fluid is placed before the patient's mouth for five or ten minutes every hour.

*Bromide of Iron* is employed by some in cases of spermatorrhea and involuntary seminal emisions, in doses of fifteen to twenty-fiye centigrammes occasionally; and, before the patient goes to sleep, in a dose of fifty centigrammes.

*Bromide of Potassium* has recently been used in cases of the sickness of pregnancy, and in cases of leaucorrhea, effecting cure in less than two months in the latter case. It is useful in summer diarrhea in infants, in doses of three centigrammes every two hours.

*Bromide of Sodium* has a similar efficacy to that of bromide of potassium in epilepsy, and proved a cure in one case of tetanus.

*Coffee* has been given in infusion in cases of infantile typhus fever.

*Conium* has been used successfully in cases of mania accompanied by muscular agitation. It acts on the motor centre, sparing the sensory tracts. Of twenty-five patients treated by this substance, twenty-two times muscular agitation subsided.

*Hydrate of Chloral* has been used in cases of nocturnal incontinence.

*Chloride of Potassium* has been used instead of bromide in epilepsy, and it asserted to be more efficacious. Dose: 3.5 grammes to 5 grammes a day.

*Copaiba* has been recommended in certain cases of psoriasis.

*Iodine* has been recommended in cases of nocturnal incontinence of the aged; one drop of the tincture every hour in water. The tincture has also been recommended in doses of ten drops in intermittent fever thrice daily.

*Iodoform* is used in chronic venereal ulcers, and much praised as an antiseptic.

*Iodide of Silver* is recommended in whooping-cough.

*Koussine* is an excellent vermifuge, and is given in the morning in doses of 1.25 grammes in a little syrup.

*Phosphorus* has been recommended in chronic skin diseases in oil, or gelatin capsules containing each from two to six milligrammes of phosphorous in oil. Acne indurata, lupus, psoriasis, and scrofulous skin diseases have been cured by such means.—*The Doctor.*—*Pacific Med. and Surg. Jour.*

*Xylol.*—This hydrocarbon is obtained from coal naphtha. It is produced by fractional distillation, until a distillate is obtained of about 140° C., boiling point; this is mixed with sulphuric acid, which dissolves xylol, forming xylol sulphuric acid. This acid is decomposed by dry distillation, and the xylol thus obtained is further purified.

Pure xylol is colorless. It has a faint odor, somewhat like benzole, but different.

The absolute purity of this remedy is important, as other analogous compounds do not possess the same peculiar properties.

*Properties and uses* not thoroughly understood, but it appears to be taken up by the blood, and acts as a disinfectant.

Used extensively in the treatment of small-pox in all its varieties. Administered in 81 cases, 40 of which had disease in its worst form; only 4 died.

I therefore conclude that, *first*, xylol produces positively better effects in the treatment of small-pox than any other remedy known at the present time.

*Second*, Xylol positively mitigates the severity of the disease, accelerates the recovery and prohibits the hitherto great mortality.

*Third*, Xylol, administered when a contagion is suspected and before the eruption of the disease, does not prevent the breaking out of small-pox, but fecilitates the elimination of the disease.

Therefore it can be relied upon and administered with complete success.

It is given in from three to five-drop doses to children. Ten to fifteen for an adult, every hour to three hours.

It is harmless, because as much as a teaspoonful at a time has been taken. The most convenient form of taking it is in capsules, already supplied by the trade.—*Eclectic Med. Jour.*

*Cholera Infantum.*—Dr. M. Taggart (*Eclectic Med. Jour.*) says of *cholera infantum*, that the treatment consists in a careful avoidance of the exciting causes. The old treatment with the comp. neutralizing mixture was, and is, a good one, but I have had far better success with the following: When called in first stages, I order—
℞.—Tinct. nux. vomica, gtts. v to x; aqua, ℥iv. Mix.

*Dose:* Teaspoonful every half hour or hour.

Alternate with the following, to control the fever and relieve irritation of the bowels and check the discharges: ℞.—Tinct. aconite rad.; tinct. ipecac., ä ä ʒss to ʒi; aqua, ℥iv. Mix.

*Dose:* Teaspoonful every half hour or hour, until the worst symptoms are controlled.

Omit the nux as soon as the vomiting ceases, depending upon the aconite and ipecac. to control the fever and discharges. Allow the patient but little fluid to drink at a time. Sponge the surface with soda water, keeping the feet warm and head cool.

When there is great prostration, stimulants must be used, as burnt brandy in suitable doses. Quinia should always be given, during the remissions, in from half to one-grain doses. Diet should be light and nutritious, consisting principally of fluid victuals.

The above directions, followed out with care, modified to meet the existing indications, will give entire satisfaction. Not one death in a hundred.

*Phthisis Cured.*—Dr. Dutcher, in *Cin. Med. Jour.*, reports a case of phthisis successfully treated as follows:

The diagnosis appeared to be obvious—tubercular softening in the superior lobe of the left lung. Treatment prescribed was: ℞.—Elix. calisary; syrup hypophosphite calcis. aa. f ℥iv; syrup sanguinarial comp., f ℥ii; solution strychniæ, U. S. D. f ʒij. M. SIG. A tablespoonful three times a day after meals.

To relieve pain in the side affected, the compound tar plaster was ordered as a counter-irritant. To mitigate night perspiration, on retiring a sponge bath composed of a drachm of acidum sulphurium aromaticum, in a pint of water, was employed, and internally, a teaspoonful of quinine, sulph., and as her appetite and digestion were good she was allowed a substantial diet.

After four weeks persistent treatment, with the above therapeutics, there was a marked mitigation in all the pressing symptoms. The physical signs and an examination of the sputum by the microscope clearly demonstrated that the softening of the tubercular matter was complete and a cavity formed. From this time she continued to improve daily in weight and strength. Her menses appeared on the first of December, and afterwards continued regular. The cough and expectoration gradually became less, and by the first of May the cavity was perfectly healed. The patient has since had a child, and at the present writing is in the enjoyment of good health.

*An Old Cancer Cure Revived.*—Dr. Brewer writes to a recent number of the *Medical Times*, that a patient of his was given by a friend, the following recipe: ℞.—Chlor. zinci, gr. viij; blood root, gr. v; starch, gr. viij. Make into a paste with honey.

The cancr was at this time nearly as large as a hen's egg. After applying the paste for two weeks, he called to see me. I found it had diminished to half its former size. I advised him by all means to continue it. After a month's use of the remedy the cancer was not larger than a dime. He continued to use it until the disease was cured.

This is the "cancer salve" of old Dr. Fell, famous some forty years ago, but long since condemned to neglect.—*Cincinnati Lancet and Observer*, February, 1874.

### REMARKS, GLEANINGS AND EXTRACTS

BY T. CURTIS SMITH, M.D., OHIO

*Quinine as a Remedy for Hay-Fever.*—By Prof. Binz, of Bonn. (*Practitioner*, April, 1874.) From what I have observed of recent English publications on the subject of hay-fever, I am led to suppose that English authorities are inaccurately acquainted with the discovery of Prof. Helmholtz, as far back as 1868, of the existence of uncommon low organisms in the nasal secretions in this complaint, and of the possibility of arresting their action by the local employment of quinine. I therefore purpose to republish the letter in which he originally announced these facts to myself, and to add some further observations on the topic. The letter is as follows:

"I have suffered, as well as I can remember, since the year 1847; from the peculiar catarrh called by the English 'hay-fever,' the specialty of which consists in its ataacking its victims regularly in the hay-season, (myself between May 20th and the end of June), that it ceases in the cooler weather, but on the other hand quickly reaches a great intensity if the patients expose themselves to heat and sunshine. An extraordinarily violent sneezing then sets in, and a strongly corrosive thin discharge, with which much epithelium is thrown off. This increases, after a few hours, to a painful inflammation of the mucous membrane and of the outside of the nose, and excites fever, with severe headache and great depression, if the patient cannot withdraw himself from the heat and the sunshine. In a cool room, however, these symptoms vanish as quickly ar they come on, and then there only remain for a few days a lessened discharge and soreness, as if caused by the loss of epithelium. I remark, by-the-way, that in all my other years I had very little tendency to catarrh or catching cold, while the hay-fever has never failed during the twenty-one years of which I have spoken, and has never attacked me earlier or later in the year than the time named. The condition is extremely troublesome, and increases, if one is obliged to be much exposed to the sun, to an excessively severe malady.

"The curious dependence of the disease on the season of the year suggested to me the thought that organisms might be the origin of the mischief. In examining the secretions I regularly found, in the last five years, certain vibrio-like bodies in it, which *at other times I could not observe* in my nasal secretion.... They are very small, and only can be recognized with the immersion-lens of a very good Hartnack's microscope. It is characteristic of the common isolated single-joints that they contain four nuclei in a row, of which two pairs are more closely united. The length of the joints is 0.004 millimetre. Upon the warm objective stage they move with moderate activity, partly in mere vibration, partly shooting backward and forward in the direction of their long axis; in lower temperature they are very inactive. Occasionally one finds them arranged in rows upon each other, or in branching series. Observed some days in the moist chamber, they vegetated again, and appeared somewhat larger and more conspicuous than immediately after their excretion. It is to be noted that only that kind of secretion contains them which is expelled by violent sneezings; that which drops slowly does not contain any. They stick tenaciously enough in the lower cavities and recesses of the nose.

"When I saw your first notice respecting the poisonous action of quinine upon infusoria, I determined at once to make an experiment with that substance, thinking that these vibrionic bodies, even if they did not cause the whole illness, still could render it much more unpleasant through their movements and decompositions caused by them. For that reason I made a neutral solution of sulphate of quinine which did not contain much of the salt (1.800), but still was effective enough, and caused moderate irritation on the mucous of the membrane of the nose. I then lay flat on my back, keeping my head very low, and poured with a pipette about four cubic centimetres into both nostrils. Then turned my head about in order to let the liquid flow in all directions.

"The desired effect was obtained immediately, and remained for some hours; I could expose myself to the sun without fits of sneezing and the other disagreeable symptoms coming on. It was sufficient to repeat the treatment three times a day, even under the most unfavorable circumstances, in order to keep myself quite free.* There were then no such vibrios in the secretion. If I only go out in the evening, it suffices to inject the quinine once a day, just before going. After continuing this treatment for some days the symptoms disappear completely, but if I leave off they return till toward the end of June.

"My first experiments with quinine date from the summer of 1867; this year (1868) I began at once as soon as the first traces of the illness appeared, and I have thus been able to stop its development completely.

"I have hesitated as yet in publishing the matter because I have found no other patient† on whom I could try the experiment.

*There is no foundation for the objection that syringing the nose could not cure asthma which accompanies hay-fever, for this asthma is only the reflex arising from the irritation of the nose.—B.

†Helmholtz, now Professor of Physics at the University of Berlin, is, although M.D., not a medical practitioner.—B.

There is, it seems to me, no doubt, considering the extraordinary regularity in the recurrence and course of the illness, that quinine had here a most quick and decided effect. And this again makes my hypothesis very probable, that the vibrios, even if being no specific form, but a very frequent one, are at least the cause of the rapid increase of the symptoms in warm air, as heat excites them to lively action."

I should be very glad if the above lines would induce medical men in England—the haunt of hay-fever—to test the observation of Helmholtz. To most patients the application with the pipette may be too difficult or impossible; I have, therefore, already suggested the use of Weber's very simple but effective nose-douche. Also it will be advisable to apply the solution of quinine *tepid.* It can, further, not be repeated often enough that quinine is frequently adulterated, especially with cinchonia, the action of which is much less to be depended upon.

Dr. Fricköfer, of Schwalbach, has communicated to me a second case in which hay-fever was cured by local application of quinine (Cf. Virchow's *Archiv* (1870), vol. li., p. 176). Prof. Busch, of Bonn, authorizes me to say that he succeeded in two cases of "catarrhus æstivus" by the same method: a third patient was obliged to abstain from the use of quinine, as it produced an unbearable irritatlon of the sensible nerves of the nose. In the autumn of 1872, Helmholtz told me that his fever was quite cured, and that in the mean time two other patients had, by his advice, tried this method, and with the same success.—*N. Y. Med. Jour.*

*Contributions to the Physiology of the Pneumogastric.*—S. Arloing and L. Tripier have published an interesting work on this subject, and have arrived at the following conclusions:

1. A section of the cord, posterior to the spinal bulb, diminishes considerably the excitability of the pneumogastric.

2. From a functional point of view, there is a notable difference between the two nerves, the right acting more energetically on the heart than the left.

3. The converse is true for the mechanical phenomenon of respiration; excitation of the left nerve modifies more profoundly the movements of the thorax than excitation of the right.

4. The arrest of the heart's action is more complete when the galvanization is applied only to the peripheric end, rather than in the course of the nerve.

5. Galvanization of the peripheric end causes arrest of the heart in diastole, while galvanization in the course tends to arrest it in systole. The pneumogastric, therefore, governs the action of the heart.

6. The movements of the heart which are produced by galvanization of the vagi are feebler than those before excitation; notwithstanding this feebleness, the pulse becomes stronger, because there is less tension in the arterial system.

7. It is impossible to embrace in a general formula the influence of galvanization of the pneumogastric on respiration.

8. Galvanization of peripheric end causes respiratory movements, probably because the pneumogastric is also sent to the periphery of the recurrent fibres.

9. Section of the pneumogastric is accompanied by feebleness in the movements of the thorax of the corresponding side.

10. Finally, it is possible that one of the pneumogastrics controls the function of digestion.—*Arch. de Phys.*, 1872. *Lyon Medical*, May, 1873. *N. Y. Journal.* E. F.

*Ulceration of Rectum; Treatment.*—It is the experience in this hospital that in ulceration of the rectum, whether it be venereal, the result of stricture, or any other cause, the application of iodoform is of more benefit than any other agent in hastening a cure and relieving pain. It may be either used in solution or as a suppository. The solution is made by adding from half a drachm of iodoform to two ounces of glycerine, and half an ounce used at a time. The suppositories are formed from butter of cacao, each suppository containing five grains of iodoform. Before using it, evacuate the bowels by means of a purgative or enema, then apply the iodoform at night.—*N. Y. Med. Jour.*

*A Dislocated Liver Mistaken for an Ovarian Tumor.*—The patient, an unmarried woman, aged twenty-four years, was the subject of a large and painful abdominal tumor, of eight month's growth, for the relief of which the operation of ovariotomy was decided upon. Upon opening the abdominal cavity, the ovary was found to be healthy, the swelling being due to an enlarged liver. This organ was free and movable, pressing upon and displacing the uterus, bladder, rectum, and other abdominal organs. — *Boston Med. and Surg. Jour.*

*Singular Death from Blood-Poisoning.*—Dr. Weigel, a surgeon connected with the military hospital at Munster, was recently made dangerously ill, the result of inoculation with morbid matter absorbed at an autopsy through a slight would in his hand. In performing an operation for his relief, another surgeon, Dr. Kruse, chanced to make a slight incision in his finger, by which means he himself became inoculated, and, after severe suffering, died.

### REMARKS, GLEANINGS AND EXTRACTS

BY A. R. KILPATRICK, M.D., TEXAS.

*The Medicinal Virtues of the Common Bur, Xanthium Spinosum.*— A few years ago I learned, through Dr. J. N. Baylor, that the expressed juice of the cockle bur would arrest ordinary hemorrhage from a cut, or from any mucous surface, such as the fauces, nares, or stomach, or vagina, if applied directly to the part. I have seen it tried as a styptic, and am satisfied with its virtues in that way. It seems to be more efficacious than acetate of lead, as it will arrest a hemorrhage when the lead has failed.

The manner of preparing it for use as a styptic is, to gather a few of the leaves and stems of the growing plant and mash them well with a mallet, hatchet, or anything convenient, and squeeze out the juice thoroughly and apply it directly to the bleeding part, or give it by the mouth in cases of hæmatemesis.

Some one sent me a copy of the "*Eclectic Medical Journal*," May and June, 1874, published by Dr. John Buchanan, Philadelphia, Penn., and in it I found a brief note from Dr. Wm. Fulton, of Centerville, Piatt county, Ill., stating that he "found it in urinary difficulties equal, and in some cases, superior to the hydrangea. I have used it in two cases of phosphatic renal calculi, when it excelled the hydrangea. In scalding and bearing down pains in urinating, it excels any remedy I have ever used."

After reading that, I addressed a note of enquiry to Dr. Fulton, asking how to prepare and administer the medicine; and in answer I received the following: "Gather the bur plants at any time when in full growth; have them well bruised and take a large manipulus, or more, for a quart of the decoction; pour on boiling water to that amount and let it draw until cool, then use in hatf cupful or whole cupfuls every hour or two, until relieved. The tea is not unpleasant and is harmless. In addition to what I stated in the *Eclectic Medical Journal*, I can now say that the decoction of the bur is also an excellent remedy in stranguary and retention of urine. I use it now with more confidence than any one remedy for urinary troubles."

Dr. Fulton proposes to put up a quantity of the tincture of the leaves this fall, for use during the winter months.

Not being content with this, I wrote also to Dr. Gid. Lincecum, of Long Point, Washington county, Texas, asking his experience with the bur. Dr. L. is now one of the oldest men in the State, and probably the oldest man belonging to the profession, and certainly better acquainted with the plants, herbs and flowers indigenous to the country, and their medical virtues, than any other in all the vast territory of Texas.

He says: "Make a pulp of the fresh leaves and apply to the skin and they will draw a blister promptly; and give the decoction of the leaves for snake bite, and it will cure it."

I hope this may direct the attention of practitioners to the virtues of this common plant, and cause them to try it still further.

*Pregnancy with Persistent Hymen.*—M. Dufour was consulted by a lady for some abdominal enlargement. On feeling the tumor, the movements of the child were apparent. On proceeding to make a vaginal examination, his finger was prevented from entering the passage by a complete circular band, which ocular demonstration proved to be the hymen. In leuchorrhœa the parts sometimes become much relaxed, and rendered thereby very dilatable, so that under some circumstances the act of coitus may be completed without any rupture of the hymen. In this case the patient said she had never suffered from the "whites," and this was substantiated by the condition found.—*London Medical Record.*

*Commercial Value of Abstract Science.*—Though the posssibility of producing alizarine by artificial means was demonstrated only in 1868, the production of Germany during 1873 reached 1,100 tons, valued at 600,000£ ($2,000,000); and it is announced that a single establishment is preparing for a production of 5,000 tons of alizarine per year.—*Ex.*

These discoveries were made by men who were persuing science for its own sake, and not because they hoped to realize money out of it; and shows again the dependence of the practical upon the purely abstract sciences.

*Active Agent of Ergot.*—Dr. A. Wernich has recently made some investigations in regard to the active principle of ergot, in the Berlin Institute. He finds that the watery extract is far more powerful than either the alcoholic or etherial extract. The watery extract, when purified by alcohol and ether, forms a mucous or slimy mass, which cannot be dried. The active agent appears to be of the nature of an acid, soluble in alcohol when pure, but insoluble when in combination with bases.—*Lancet—Amer. Jour.*

---

*Lime-Water in Stings of Bees.*—M. Dauverne states, as the result of numerous trials, that the pain and suffering caused by these may be immediately assuaged by the application of lime-water; a remedy which may always be prepared at once by the aid of a little quicklime and a glass of water.—*Med. and Surg. Reporter.*

### REMARKS, GLEANINGS AND EXTRACTS

BY S. M. BURNETT, M.D., OF TENNESSEE.

*Ether and Chloroform—Their Relative Dangers.*—In the *Practitioner* (London) for April, Dr. Hake gives the results he has witnessed the physiological labratory of Prof. Schiff at Florence, of giving ether and chloroform to animals. Prof. Schiff has found "that etherization pushed to the very last stage of insensibility is never dangerous to life so long as one maintains the act of respiration, and even if one press the inhalation of ether yet further, so that the respiratory movements cease, or in other words the appearance of death is complete, life is never menaced, if only, at the moment of paralysis of the thoracic walls, inhalation be interrupted, and a species of artificial respiration be be immediately commenced, by means of periodic compression of the thoracic walls themselves.

"Chloroform has been preferred to ether because it acts more quickly, and its use is more agreeable to the patient. But chloroform has a paralyzing action much greater than that of ether, and in like manner, at least in man and the mammalia, generally has a special influence on the heart and vessels. If chloroform be pushed so as to produce a considerable weaking of the respiratory movements, the interruption of the inhalation may, in a majority of cases, lead to the re-establishment of respiration, and afterwards of sensation; but sometimes, a short time after the commencement of inhalation, the force of the circulation is so enfeebled that it no longer renews fast enough the blood to the lungs....If the action of chloroform be paralyzed until respiration ceases, we are not even sure of being able to revive the individual after having re-established the respiratory movements, for these often again cease, owing the disturbance of the circulation, whilst the same movements, if restored after the inhalation of ether, become *always* more frequent in the individual when left to himself."

It is Prof. Schiff's opinion "that chloroform should be banished from practice as anæsthetic agent, except in cases in which an extraordinary resistance to the effect of ether shows itself, in which instances it might be allowed to mix a little chloroform with it, in order to produce the commencement of anæsthesia."

*Stricture of the Urethra.*—In concluding a long and able paper on "Urethrotomy, Internal and External," Dr. F. N. Otis makes the exertions that strictures, as ordinarily met with, are absolutely within the reach of curative measures; that if completely divided, and this division maintained until healing of the parts has occurred, no re-contraction can ever take place, and that division of the stricture is not more hazardous, to say the least, than rapid, permanent, or even temporary dilatation.

He says stricture may be present before difficulty in urinating occurs; it is always present when gleet is present—gleet, as a rule, meaning stricture. Dilatation of the structure is, at best, but a temporary expedient—valuable in close stricture when urination is interferred with, and dividing instruments cannot be introduced; but dilatation is not only without permanent value, except in such cases, but it is pernicious, inasmuch as while it is never curative, it takes the place of curative measures. He concludes by asserting that nothing short of complete division of strictures can ever result in radical cure.—*Western Lancet.*

*Unreliability of Elixirs.*—The great furor among physicians for palatable medicines has had the effect to bring on the market some very inert preparations. We clip the following from the *Tennessee Pharmacal Gazette*, as bearing on this point, and suggest to practitioners before prescribing elixirs to consider whether the ingredients are incompatible, or their virtues are not destroyed in process of manufactuare:

G. W. Albers (Tenn.) sends the following formula for *Elixir pepsin, bismuth and Strychnia:* Take of Ammon cit. bismuth, 144 grs.; pepsin, 720 grs.; strychnia, 2 grs.; ext. vanilla fluid, 2 ozs.; syr. cort. aurant, 4 ozs.; Sp. vini gallici, 4 ozs.; aqua, 8 ozs.; acid. mur. C. P., Q. S. Misce scundem artem.

This preparation is extensively sold by many responsible manufacturing pharmacists, aud prescribed by physicians with the vain délusion that they are administering a palatable elixir, containing the virtues of pepsin and bismuth. It is a well known fact that acid precipitates the bismuth and the spirit impairs the activity of pepsin.

This is one of those officinal preparations, whose mode of compounding is unknown to physicians. They would never prescribe it, if aware of its chemical incompatibilities.

*Painless Method of Cauterizing with Nitric Acid.*—It is found in Charity Hospital, N. Y., that chancroids can be cauterized with nitric acid without severe pain, by first applying pure carbolic acid to the sore. The carbolic acid serves as a local anæsthetic, and prevents the nitric acid from causing pain, which is not easily borne by the patient.—*N. Y. Med. Jour.*

Over two years ago, Dr. Bailey, of this place, told us he had been using carbolic acid before cauterizing chancroids, and that the pain was thereby almost edtirely obviated. Since that time we have ourselves been using it, and have it to answer most admirably. In some cases the patients say they experience no pain at all from the nitric acid cauterization.

### REMARKS, GLEANINGS AND EXTRACTS

BY LOCAL EDITORS.

*Typhoid Fever—Treatment.*—Dr. Little, (*Dublin Journal of Medicine,*) says: Milk should be the chief article of diet in enteric fever. Thirsty patients sometimes object to its mawkish taste, and in that case ice should be added, and a little lime-water in cases where it returns curdled. Junket or renneted milk, given before it has separated into whey, and curd, rice milk, custard, baked custard in small quantities, rusks and hot milk, and blanc-mange, generally afford sufficiently varied ways of giving milk. Freshly made chicken jelly is less liable than beef-tea to increase the abdominal symptoms, in those cases where milk even with lime-water disagrees; but Dr. Little finds that this is a very rare occurrence, and when encountered is usually in a person chronically dyspeptic. For years he has made the administration of two or three cups of really good tea or coffee, between day-break and two in the afternoon, a regular part of the treatment in every case of fever, unless there was in the state of the nervous system some evident contra-indication. This he recommends in consequence of the well-known observations of Dr. Parkers on the effect of coffee in increasing the elimination of urea in fever; and Dr. Little finds that both it and tea lessen drowsiness and prostration, and increase the secretion of urine. Once or twice in the day they máy be given, poured upon a well-whisked egg, and thereby an additional means of nourishing the patient is obtained. Dr. Little considers that alcoholic stimulants in any quantity are seldom needed. Cold baths he thinks serviceable: three, or at most four, may be given in the twenty-four hours. In severe cases he has used them with great benefit, where cooing and wheezing râles exist in the chest, and where deficiency in the percussion-resonance posteriorly and muco-crepitus indicated postural stasis in the lungs, but not when there was hemorrhage from the bowel, or such pain as to justify the fear that peritonitis existed. Where there is slight chillness in the extremites after a bath, and shivering, this indicates that it should not be a prolonged one, but does not forbid its use. Twice Dr. Little has considered it unsafe to continue the baths—once because a marked shivering followed, and once because the patient was alarmed by it. In cases of the disease running a mild course, it is not necessary to have more than one bath in the day, at the height of the usual evening paroxysm of fever. By a dietary such as has been described, and the systematic employment of baths, the severity and danger of enteric fever may be greatly diminished, and the occurrence of any of the serious accidents incidental to the complaint rendered very rare, but the period of duration is not shortened. Besides these means, Dr. Little has found others beneficial under certain conditions. When during the first eight days the face is flushed, and there is headache, a high temperature, and a thickly coated tongue, and when the evacuations, three or four in the twenty-four hours, are neither very large nor very liquid, a dose of calomel, from four to six grains, perceptibly lessens the heaviness of the fever. He has sometimes given the calomel a second time after an interval of a day or two, but never oftener. In enteric fever it is not uncommon to find a patient lying on his back, perceptibly impeded in his breathing, his abdomen tumid and projecting, but markedly tender; and on inquiry it will be found either that the bowels have not acted for twelve hours, or that, though the stools are frequent, only a very little fæcal matter with wind passes each time. In these cases much relief may be obtained by giving a draught containing two drachms of castor oil with one or two of turpentine. Poultices and fomentations he has not found useful. By keeping patients rigidly to the diet mentioned, it is not found necessary to give medicines to check loosenees of the bowels: when it is necessary to interfere, the most useful remedy is a pill containing one-sixth of a grain of carbolic acid, one-sixth of a grain of opium, and three grains of bismuth. Another remedy is sulphuric acid. Hemorrhage from the bowels is rare when milk diet and cold baths are employed; when it occurs, gallic acid, a scruple every second or third hour, and turpentine, were the remedies upon which he relied. Since ergotin has been shown to possess the power of arresting hemorrhage, administered hypodermically, Dr. Little has tried it in one case successfully. There is a group of nervous phenomena sometimes present in typhoid fever, for which the remedy is a full dose of quinine. For delirium and wakefulness with severe headache, cutting the hair and leeches are the remedies. Nauseau and persistent retching may be relieved by an emetic of ipecacuanha or ice, or a draught containing ten grains of bicarbonate of soda, ten grains of carbonate of bismuth, and four minims of prussic acid. Scantiness of urine requires dry cupping of the loins, and the internal use of the salts of potash and spirit of nitrous ether. Indications of pulmonary congestion, which are sufficiently common in enteric fever, are best relieved by a turpentine stupe.

*Phlegmasia Dolens.*—Dr. A. P. Ten Eyck, in a paper read before the Medical Society of New York (*Phil. Med. Times*, May 17, 1873) in regard to Treatment, he said he supported the system and administered anodynes to relieve pain. Iron and quinine were used as a tonic; the bowels were occasionally regulated with Seidlitz powders. As local applications he used a weak solution of carbolic acid, applied hot, to the first limb affected, but to the second limb attacked he used sulph. ferri to the ounce of water, as hot as could be borne to the limb, it being bandaged from the body to the toes. This seemed to give better satisfaction and to give more relief than anything else, the only objection to its application being its permanent stain to the clothing.

Dr. James S. Bailey said that he had seen an unusual number of cases of this disease, and that four cases had occurred in his own practice, in the first seventy-five cases of midwifery that he had attended, in the year 1871. In three cases it only appeared in one leg; in one case in both legs, first one and then in the other: one of the women was a primipara, the other three multiparæ. The woman in whom it occurred in both limbs was delivered of twins. She died in the course of three months, from exhaustion. He regretted that he could not obtain the consent of the friends to a post-mortem examination.

The plan of treatment adopted was to support the system, keep the bowels regulated, give anodynes to relieve pain and procure rest, and to wrap the affected limb in flannel cloths wrung out of hot water and applied as warm as could be borne, and this dressing covered with oiled silk. These applications were continued until all signs of inflammation had subsided.

Dr. S. H. Freeman remarked that he had occasionally in his practice met with cases in females of lymphatic temperaments with light complexions, and had used spirits of turpentine freely, but with care, to the affected member. In one case, occurring not long since, the nurse had used it rather lavishly, and it had produced vesication, after which he had wrapped the limb in cotton batting and covered this with oiled silk. The case did well, and recovered in three weeks.

Dr. F. C. Curtis said that Prof. Thomas, of New York, recommended a vesicant along the course of the vein, at one side of it.

Dr. E. A. Davis said the treatment depended upon the general condition of the system. In case of plethora and fulness, with violent inflammatory symptoms, he would not hesitate to bleed; but, in an opposite condition of the system, quinine, with other tonics, might be early necessary. The local application of the hot infusion of hops had been found very serviceable in his practice, followed later by stimulating embrocations.

*Chronic Diarrhœa.*—Dr. Scudder, (*Eclectic Med. Jour.,*) says: Epilobium is one of the best remedies we have to cure chronic inflammation of the mucous membrane of the intestine. It allays irritation, rectifies wrongs of innervation, stimulates normal functional activity—does just that which seems to be necessary to effect a cure. It acts very slowly, but quite surely, and will cure most obstinate and old cases. I use it in infusion, ℨij. to boiling water, ℥iv.; a tablespoonful every one, two or three hours. It cured hundreds of cases of "camp diarrhœa" during and after the war. Let me say further, that Epilobium stands first in the list of remedies for diarrhœa in typhoid fever, especially with severe ulceration of Peyer's glands.

Liquor Bismuth stands second in the list of empirical remedies, and may be used with most satisfactory results in some cases. I should advise its employment when the person suffered also from *irritative* dyspepsia or chronic gastro-enteritis; in these its action is very decided. The Liquor Bismuth is much to be preferred to the insoluble Sub-Nitrate.

Hamamelis would probably be classed third in this list, and used in the ordinary dose of half a teaspoonful of Pond's Extract every four hours, or the infusion taken freely, or the ordinary tincture, would sometimes give good results.

The White Liquid Physic has had considerable use in these cases, and has been "quite highly esteemed"—to use a popular medical expression. The dose is small, a teaspoonful every three or four hours, "to keep up a gentle influence upon the intestinal tract."

In so far as the chronic wrong of the intestinal canal is concerned, these are our best remedies, and I do not know that I can point out any special indications that will advantage the reader. The Epilobium might be specially indicated by tormina, or by accumulation of gas in intestine. The Hamamelis would be especially indicated if there was sense of fullness in the pelvis, and relaxation of pelvic and perineal muscles. The Bismuth would be selected if the tongue was full in the center, with a whitish, slippery, mucoid coat, but better by the conditions of gastric irritation.

Podophyllin triturated is a valuable remedy when there is abundance of mucus in the discharges, or muco-pus. We may triturate with Bismuth in some cases with advantage; in others it acts better if we add the bitter principle of Hydrastis.

Arsenic in small doses is a very valuable remedy in some cases, but not in a large number, and it must be specially indicated if given.

I like the wet pack, used at night, either of water, vinegar, or water accidulated with Muriatic Acid—the last two are favorites. Using the Acid we take a flannel bandage, wring it quite dry, and pin around the abdomen on going to bed, with a dry towel over it. In the morning the lower part of the trunk is thoroughly sponged with cold water and briskly rubbed with a towel.

*Treatment of Acute Articular Rheumatism by Chlor-Hydrate of Trimethylamine.*—Dr. Jean Paul Bonsieur, in *Philadelphia Medical Times*, says: Since the introduction of this drug into notice by Dr. Dujardin Beaumetz, I have had occasion to witness its excellent effects.

Case.—X., Compositor, æt. 34, attacked March 7, after chill and fever with articular pains. Never suffered from rheumatism; no hereditary taint. On the 8th I diagnosed acute articular rheumatism. All the points of the superior and inferior extremities were swollen and extremely painful; the patient being incapable of the slightest movement. The pulse was full, strong, and frequent—89 per minute; perspiration very abundant. The following prescription was ordered: ℞ Aq., parts (by weight) 100; aq. menth. pip., parts (by weight) 40; syr. aurant. cort., parts (by weight) 30; trimethylaminæ chlorhydrat., part (by weight) ½. M. et Sig. Exhib. coch. mag. om. bis hor.

March 9.—Insomnia, abundant perspiration, urine increased; pulse full, strong, 69; articular pain less intense, disappeared from some joints; swelling and redness still persistent. The patient takes the medicine with pleasure, the taste being masked by the peppermint-water, though the odor still persists.

March 10.—Slept four hours during the night; slight moisture on the skin; pulse full, large, 60 to the minute; tongue clean; appetite good; the articular pain has entirely disappeared, together with the redness and tumefaction. The patient is now able to move his limbs without pain, and with the greatest facility. The medicine is well received by the system; no gastric trouble. Continue prescription every four hours.

March 11.—The amelioration continues; the patient keeps his bed, in order not to compromise his cure; pulse large, 51 to the minute; skin slightly moist; no cerebral or cardiac involvement; appetite good; urine abundant; sleep perfect. Continue the prescription through the following day. Discharged.

*Internal Metritis.*—In a clinical lecture, Prof. Getchell, remarks: Nitrate of silver is highly recommended; but, while I prefer it for cervical endometritis, I very seldom use it above the os internum. I have invariably been disappointed with it in the treatment of internal metritis, while with iodine I have had every reason to be satisfied. The formula I use is—℞. Potass. iodidi, ʒss; iodinii, ℈iv; glycerinæ, ℥j. M. The application may be made every eight or ten days; it gives but little pain, and the patient is required to keep her bed but one day. The length of time required to relieve these patients of course depends very much upon circumstances: while some will menstruate without pain after a few applications, others will require treatment for a much longer time before relief is experienced, and in some cases you will be obliged to resort to more powerful applications. After applying the above a number of times, if there is no improvement, I then use nitric acid; there is no danger in applying this powerful caustic to the uterine mucous membrane; at any rate, I have often applied fuming nitric acid without the slightest bad effect, and its remedical power in these cases is remarkable. In using the nitric acid, care should always be taken to protect the cervical canal; this may be done by passing the probe, with the wisp of cotton saturated with the acid, through a glass tube, a piece of a large-sized gum catheter, or through the ordinary uterine speculum. The nitric acid should be applied at longer intervals, and the patient must remain at rest in the horizontal position till all fear of inflammatory action has passed. It may appear singular to you that an inflammation of a mucous membrane alone should remain for so long a time and cause so much discomfort, and also that the parenchyma is not involved; but if you will recall your anatomy for a moment you will remember that the lining membrane of the uterine cavity is totally unlike the mucous membrane that is found in other parts: so thick is it that it makes up nearly one-fourth of the uterine wall, and when once it becomes the seat of chronic inflammation there is little or no hope that it will subside spontaneously.

*Ergotine—its Use Subcutaneously in Hemorrhage.*—D. Drascher believes that ergotine must be regarded as the best of all hæmostatic agents for hypodermic use. On two persons, in robust health, the injection of from one-half grain to two grains was uniformly followed by a diminution in the pulse of from four to six beats, and the sphygmograph showed a simultaneous contraction of the calibre of the vessels. The temperature was slightly raised, respiration normal, no influence upon the urine, and the health was quite undisturbed. Some local irritation was caused, but usually passed away soon. Dr. Drasche has employed this method chiefly in tubercular hæmoptysis in doses of one-half to one and a-half grains, and in most of the cases the efficacy of the ergotine was very prompt, when other hæmostatics had previously been tried without success; in others, although less speedy, it was still successful, and in one case only out of nine did it fail. In two cases of epistaxis the effect was very speedy, and the dose required very small. It has also been used with marked advantage in hæmatemesis, intestinal hemorrhage, and hemorrhagic scorbutus. The best vehicle is glycerin, containing five grains in one drachm, and the injection is best made in the region of the pectoral muscles. When the injections have been long continued, or have been too strong, tingling of the finger-ends and cramps of the hands have been sometimes complained of.—*Medical Times and Gazette.*

*Treatment of Adenitis by Collodion.*—The author, Dr. Vogelsang (Biel), has treated a woman for a long time time for a submaxillary adenoma, the size of an egg, without any satisfactory result. Tincture of iodine, iodine ointments, cataplasms, bandages, etc., had all been applied in vain. The skin in consequence of their use had become red and inflamed.

Another physician had used iodine injections with the same result, when at last recourse was about to be had to the knife.

At this juncture, the patient was advised to cover the tumor with a thick layer of collodion. Eight days after this application was made, the tumor had disappeared.

With this result before him, the author determined to adoopt this treatment regularly in similar cases. He explains its action as due to a compression analogous to that exercised in orchitis by the compression of the strips of diachylon plaster.

The author next had to treat an adenoma behind the ear, in a well-formed young girl of twenty years of age; all the old methods had been exhausted in vain. In this case the iodine was added to the collodion, and after several applications (the first of which seemed to increase its size) it gradually gave way.

The best method of treatment is first to paint over the surface of the tumor with the tincture of iodine, and then coat this over with a layer of pure collodion, which should be daily renewed.

The author is not able as yet to state whether this method may also be applied to scrofulous swellings, but he thinks it worthy of trial.—Memorabilien ii, 1873—*France Medicale*, May 14, '73—*Clinic.*

*Cancrum Oris.*—Dr. J. G. Miller (*Kansas City Med. Journal*) having a case of cancrum oris, treated it as follows: Nourishing drinks, stimulants, tonics and antiseptics, were ordered to be pushed to the uttermost extent that the system would tolerate. We also removed as much of the dead tissues as we possibly could get away; then applied a saturated tincture of iodine, prepared by putting as much iodine into the compound tincture as it would dissolve. Wherever we could apply the medicine thoroughly, the further progress of the disease was speedily arrested.

*Dental Caries.—Tannin.*—An excellent application is creosote mixed with a little collodion. By this means a gelatinous consistency is obtained in the agent employed, which, forming a varnish over any orifice in the carious tooth, prevents the access of air to the sensitive parts. A strong solution of tannin in camphorated spirits of wine is also of use.—*Eclectic Med. Jour.*

*Uterine Pains.*—Prof. W. T. Lusk, (New York Academy of Medicine, *Med. Record,*) says: In the treatment of a pathological weakness of pains, one or two courses are open to the obstetrician: he may either effect delivery by resorting to a judiciously selected operative procedure, or by applying the whip and the spur he may seek to compel the uterus to perform its proper function.

Both from training and inclination the author prefers in most, if not all cases, to adopt the first alternative. Yet he is not quite ready to deny that ergot may be profitably employed at even an early stage of labor. He has seen small doses of ergotine given in Prague Hospital, with the view to awaken such a degree of uterine activity as would suffice to bring about the moulding of the head in a moderately contracted pelvis. The ordinary formula consisted of: ℞. Ergotine, ℈j.; tinc. cinnamomi, syr. aa℥j. M. et sig.: Teaspoonful every three or four hours.

In these cases it appeared to produce good results, and certainly did no harm, unless perhaps by leading to the postponement of a more vigorous line of treatment.

He mentioned a plan for supplementing weak pains that has been long employed by practitioners, for which two rules have been more recently formulated by Kristeller, consisting of regulated pressure made through the abdominal walls upon the fundus uteri. This method, to which Kristeller has given the name of *expressio fœtus,* in some cases enables us to bring about delivery without the employment of an extractive force. Yet the number of such cases is limited, and its principal application will always, it is likely, be found as a support to other operative procedures belonging to midwifery. Those who are not accustomed to resort to this plan, will be astonished upon trial to find how much a scientifically applied *vis a tergo* facilitates forceps deliveries, and extraction following versions.

*On a New Method of Determining the Presence of, and Recovery from, True Ringworm.*—By Dyce Duckworth, M.D.—The author called attention to the action of chloroform upon the infected hairs in cases of tinea tonsurans. It was shown that this agent caused the hairs to become white or slightly yellow in color, and thus to be distinctly mapped out and easily distinguishable from surrounding healthy hairs. The causes of the change were briefly discussed, and the particular phases of the disorder suitable for this application were pointed out. The effect of chloroform on patches of favus, tinea versicolor, melasma, and alopecia areata was likewise discussed. It was shown that no other reagent, so far as was know, possessed the peculiar properties of chloroform in affecting parasitically diseased hairs.—*Transactions British Association, British Med. Journal.*

*A New Treatment for Tape-worm.*—In March last, J. M—— applied to me to rid him of a tape-worm. He had been under treatment previously; had taken kousso, malefern, and pumpkin-seed, without cure. I gave him large doses of turpentine, with no effect save to excite some inflammation of the stomach. In August, the man applied to me again, not so much to be rid of the worm as for relief from the gastric troubles excited by the animal. After these had passed off, I gave, with the usual precautions, a half-ounce of fluid extract of malefern, but still without the hoped-for effect.

The idea then struck me, if small quantities of "carbolic acid" are so pernicious to leeches, is it not probable that the drug might be used against a tape-worm. Accordingly, having purged the patient, I administered six grains of "carbolic acid" in a half-pint of water, four times a day. After two days of this treatment, as only a few joints of the worm had been voided, I changed the vehicle from a liquid to a pill form. Powdered extract of liquorice was used, and each five-grain pill contained two grains of "carbolic acid;" of these the man took one every hour, and a purge of rhubarb and jalap every morning. He soon began to pass large fragments of the worm, and on the third day the head and about four feet of the body of a tænia solium. He had taken thirty-five pills without inconvenience of any kind. He got well quickly, has remained well since, and I am satisfied is cured. I have since thought that the pills would have been improved by a delicate coating of paraffine; the object being, of course, to cause the pill to pass through the stomach unchanged into the intestine, carrying with it the "carbolic acid" in direct contact with the worm.—*Medical Record.*

*Varicose Veins.*—Dr. J. H. Stearns, (*Northwestern Medical Journal*), writes: Many persons are afflicted with ulcers on the legs, the result of varicose veins.

The method I have employed is to use a needle and silk thread, beginning at the lowest practicable point of the enlarged vein. I carry the point through *under* the vein, drawing the thread well through, then return through the point of exit *over* the vein and out at the point of entrance, and tie over a piece of quill or other suitable support.

Treat the higher point of the vein in the same way. In a few days the silk may be cut. Very little inflammation usually results and the vein will be obliterated, the circulation seeking other veins to convey the returning blood, and with the removal of the cause the ulcers will heal with the most simplest dressings. As no large nerves accompany the superficial veins there is no danger in this direction.

*Cholera Infantum.*—Dr. Edward R. Palmer says (*The American Practitioner* for August): "Previous numbers of *The Practitioner* have contained articles highly commendatory of the calomel and of the bismuth and pepsin treatment of cholera infantum. I have tried both, and am fully convinced of their inferiority in this disease to the creosote and lead treatment. In the treatment of a large number of cases during the last three summers, but one has proved fatal, and that one was in a state of complete collapse before it was seen. The formula used is as follows: ℞. Mucilaginis acaciæ, ℥ss; liquoris calcis, ℥iss; creosoti, gtt. iij; plumbi acetatis, gr. xvj. S. A teaspoonful to every one to three hours.

"One thing proved is the innocuousness of the acetate, no symptom of lead-poisoning having in any instance resulted. A former professor of chemistry in this city was in the habit of asserting that sugar of lead might be given in scruple or half-drachm doses without any toxic effect, which seems, though contrary to general opinion, exceedingly plausible.

"Egg-water—the white of one egg to each gobletful—is given in conjunction with the above treatment. By adding a good-sized piece of bicarbonate of soda to each glassful, the retention of this drink by the stomach is almost assured.

"Finally, in the above, reference is had to those cases only which are strictly cholera infantum, enterocolitis being excluded as a disease which during its protracted course usually needs a variety of treatment. Here too, however, creosote and lead will give great satisfaction."

*Treatment of Endemic Dysentery.*—Dr. F. Walton Todd, of Stockton, in the report of the State Board of Health, referring to the dysentery which is often endemic in the valley of the San Joaquin, alludes to the treatment in the following terms: Several, myself among the number, think that in heroic doses of ipecacuanha we have a most invaluable remedy. Of the two hundred and fifty-one cases reported, I treated twenty-two, without the loss of a single case; and I ascribe this good result, in a great degree, to the use of ipecac., in doses of from five to twenty grains, according to the age of the patient, given every five hours, preceded by laudanum, and sometimes a mustard cataplasm. Some of these cases obstinately persisted, after a full and fair trial of the remedy—as has been the case in former years—but, it failing, a resort to castor oil and turpentine with opium and sometimes quinine, has accomplished what the ipecac. failed to do. The stomach soon acquires tolerance of the remedy in large doses, and I have been much surprised to find how soon alvine evacuations have followed upon its use.—*Pacific Medical and Surgical Journal.*

*Chloral in Nocturnal Incontinence of Urine.*—By Dr. William Thompson, Consulting Surgeon to Peterborough Infirmary.

1. We must bear in mind that chloral is not always certain in its action.

2. Only give at night, the patient having fasted for two hours previous to going to bed.

3. Give it in full doses, and when in bed.

4. Let the patient have as little fluids as possible; beer and spirits to be entirely prohibited.

5. Not to be continued longer than a week or ten days; if benefit is not derived within that period, the case is either not one of incontinence depending on habit solely, or the peculiar idiosyncrasy of the patient prevents the beneficial action of the chloral.

I might say much on the employment of chloral in combination with other remedies when the incontinence depends on some specific cause, such as disease of the bladder, urethra, or kidneys, or from reflex irritation from ascarides, from constitutional debility, aræmia. In all these cases it may prove useful in combination. But in fifteen simple cases treated by me during the past three years, it has proved alone a perfect cure in all in less than a week.

In conclusion, I would add that it is my belief that when chloral is employed in this disease, with the rules advised, it will prove highly satisfactory both to the physician and patient, as it enables us to cure speedily a disease the mere discussion of whose symptoms is a source of humiliation to the sufferer.—*Lancet.*

*Sick Headache.*—Dr. S. Rhoades (*Med. and Surg. Reporter*) says: "In the last twenty years I have permanently cuied many cases of sick headache, by giving, three times a day, ten drops of tincture of nux vomica, and continuing this treatment from two to six months. When a paroxysm of pain is threatened, give an extra dose or two of twenty drops each. If, in spite of this, the paroxysm come on and be severe, with great nausea, administer a mild emetic, and follow that with a dose of bicarbonate of soda and comp. spirits of lavender. If morphine be used at all, combine a small dose of it with a large dose of quinine.

Persons subject to sick headache often have other ailments, which require remedies appropriate therefor; but, for the headache itself, I prize the nux vomica above all the rest of the Materia Medica. When the patient objects to the bitter taste of the tincture, I give instead of it, a sugar-coated pill of the extract, or of strychnia.

Opium and its preparations have their proper but limited use in this, as in many other diseases. Their excessive use by some physicians of the present day is an error, which ranks with the blood-letting of forty years ago."

*New Method of Treating Fractures of Clavicle.*—M. Broca, having a case, treated it as follows: He placed the limb in a semiflexed position behind the back, when the most perfect confluence of the fragments occurred. The arm was fixed in this position by a bandage, and kept in it for eighteen days. At the expiration of this time the bandage was removed and the arm set at liberty. When it was found that the parts were sufficiently consolidated to prevent any likelihood of displacement, the limb was brought forward and kept immovable in a sling for a few days longer. This method of treatment has been regarded as excessively painful, but in this instance the patient only complained of the inconvenience and pain for the first twenty-four hours. The result was so good, says M. Broca, that had the patient been a lady, she might have worn a low dress without any disfigurement being observable. M. Broca does not think this plan applicable to all cases, since it compels the patient to sleep on the opposite side; but he agrees with Malgaigne in believing that in some fractures of the clavicle, the broken ends of the bone can only be brought into opposition by placing the upper extremity in special and peculiar positions, which may be quite different in different instances.

*Papilloma of the Umbilicus.*—The removal, by caustic, of a large and deep papilloma of the umbilicus, by Prof. Rizzoli, is noticed in the *Nuova Liguria Medica*, January 20, 1873. Papillomas and cancroids of umbilicus are quite rare, and their removal by the knife has usually resulted fatally. Rizzoli cites several cases occurring in his own practice.

The patient was a female, fifty-one years of age, of good constitution, having an ulcerated papilloma of the umbilicus. The tumor did not extend beyond the ring. A paste was made of eight grammes of chloride of zinc dissolved in alcohol, and mixed with eight grammes of wheat-flour. The surface of the papilloma was covered with this paste, and charpie and a bandage applied. The pain and inflammation occasioned were not very severe, and after nine days the papilloma was reduced to an eschar, which became detatched, leaving a clean, conical wound, with its base presenting externally. The acuminated portion corresponded precisely with the umbilical ring, which remained intact. The wound healed nicely, leaving a depressed cicatrix.—*N. Y. Med. Jour.*

*Diphtheria.*—Dr. Ed. L. Duer, of Philadelphia, strongly recommends the treatment of diphtheria by calomel and soda—$\frac{1}{8}$ to $\frac{1}{4}$ grain of the former, and 5 to 10 grains of the latter, in combination, according to age, every 2 hours, with brandy and nutritious food, as required. No local application necessary. His results have been uniformly favorable. His article appears in the *Obstetrical Journal* for July.

*On the Induction of Abortion for Excessive Vomiting.*—Dr. M. M. Pallen, in the *St. Louis Med. and Surg. Journal* for September, says: "The rules which guide me are these: if the patient cannot retain anything on her stomach whatever, vomiting up even cold water; if she is daily losing flesh, showing that she really does not retain any portion of the food taken, and all the known remedies in such cases have been tried and found inefficient [including electricity, which has promptly relieved cases which resisted all other remedies; also, quinine in ten-grain doses per rectum]. If there be insomnia and restlessness, and if the pulse is under one hundred, then the operation ought to be performed. But if the pulse is one hundred and twenty or more, and there are tinnitus aurium, delirium, and dimness of vision, it is too late—she will die. I have done it five times at the request of others, but every patient, as I anticipated, died."

*Anti-Neuralgic Snuff of Tobacco and Quinine.* (*Revista Clinica Bologna*, March, 1873, and *Lyon Medicale*, May 25, 1873.)—The mixture of tobacco and quinine is made in the following proportions: Citrate of quinine 50 centigr., tobacco, well fermented and irritant, one grain. Dr. Francesco Scriffignano employs this preparation principally in cases of intermittent facial neuralgia. He advises the use of several pinches of the snuff during the accession for three consecutive days. This method of administering quinine is analogous to the hypodermic method. The medicine acts almost directly on the diseased nerve by means of the ethmoidal branch of the nasal filament of the opthalmic, a branch of the fifth pair. The first access after the use of the remedy is notably much diminished in intensity, the following one is still more so, and, after the third, or at most the fourth day, the pain does not reappear.—*N. Y. Medical Journal.*

*Suppression of Urine in Typhoid Fever.*—Dr. Eaton, (*Indian Med. Jour.*) says he was called in consultation to a young lady suffering from a relapse of typhoid fever, so that she was worse than at any time previously. Had had convulsions followed by coma for several hours. All the symptoms of typhoid fever were present in a marked degree. The quantity of urine secreted for several days previously was lessened, to which cause was ascribed the relapse. The following was then prescribed: ℞. Acetate of potash, ℥ss; fl. ext. buchu, fl. ʒj; spts. nitre; camphor water, aa. ℥if. Sig. Tablespoonful every four hours.

The condition of the patient was improved by next morning; no symptoms of convulsions noticed after the urinary become free. The patient recovered in the usual time.

*The Role which the Vehicle should play in Hypodermic Injections.* (*Repertorie de Pharmacie*, 1872, No. 2.)—In this article Dr. Constantine Paul speaks first of distilled water, and recalls the fact that the injections of this liquid, made by M. Potain in cases of articular rheumatism, have frequently quieted the pain; that lumbar myosalgia is rapidly cured by them; that they were successfully used in the treatment of hepatic colic, neuralgia, etc. Also, that an injection of water, made in the region where a blister is to be applied, facilitates the action of the latter and suppresses the pain. Finally, Dr. Paul recommends the subcutaneous injection of water in cases where it is necessary to attack the element of *pain*. The quantity of water to be used is from half a gramme to a gramme. Care should be taken to bury the point of the canula in the cellular tissue, and to empty the syringe slowly and without violence.—*N. Y. Med. Jour.*

*Buttermilk in Mortification.*—Dr. T. J. Hutton says: We first used it as a local application at the suggestion of our esteemed friend, Dr. J. T. Carpenter. A boy, aged sixteen, had his arm severely bruised and lacerated, having been run over by a railroad wagon. The skin was almost entirely gone from the wrist to the elbow; the muscles were bruised and torn, and the radius scraped by the flange. Mortification followed, and despite poultices, nitric acid, and nitric acid lotion, nitrate of silver, and everything else, vesicle, bleb, sphacelus followed vesicle bleb and sphacelus inch by inch. The Doctor was called in consultation, and fully agreed with us that there was little or no hope of saving the limb, and that we should amputate next day. It was in the country; the dressing was used up, and buttermilk was resorted to for the night. So marked was the improvement next morning that this dressing was continued, and the boy made a good recovery.

*The Pharmacy of Ergot.*—At the recent meeting of the American Pharmaceutical Association ergot and its preparations was the subject of a volunteer paper by Dr. E. R. Squibb. In this the writer reviewed first the character of the ergot now found in market, stating that it was largely composed of other ergotized substances, such as grasses, barley, wheat, etc. He alluded to the fluid extract of the present U. S. P. as being a very great mistake, as it would not produce such a reliable preparation as that made by the U. S. P. of 1860. Dr. Squibb spoke of its recent administration hypodermically, and stated that he has prepared for that purpose a Solid Extract of such strength that one grain represents six minims of Fluid Extract, and that it has met with great success.

*Chronic Vomiting in Children—Tubercular Meningitis.*—Dr. D. N. Kinsman (*Kansas City Med. Jour.*) says: As to the treatment of chronic vomiting, first, we must modify the food. Lime-water and milk should be given, and when this disagrees, I have often found that beef broiled rare would be retained and digested. So also animal broths are of service when milk in any form is injurious.

For medicines: Bismuthi sub nit. in five-grain doses, or two-drop doses of vini ipecac., are often beneficial, but in my experience the elixir chloroformi or Hartshorne's cholera cure is the best remedy which can be given. The formula is as follows: ℞. Chloroformi, spts. camphoræ, tinct. opii, spts. ammoniæ, a a ʒiss; alcohol, ʒij. Mix. To the above I add ol. cinnamoni gtt. viij., and ol. amygdalæ amaræ gtt. viij.; of this mixture I am accustomed to give six or eight drops in a little water every hour till relieved.

Counter-irritation by means of mustard poultices over the abdomen, spts. of camphor, or chloroform liniment, are also beneficial.

When the patient is depressed I use coffee, which I have found a very useful stimulant, or the best old brandy.

Eustace Smith speaks favorably of inunction with oil, claiming that it adds to the nutrition of the patient.

*Oil in Surgery.*—The good Samaritan dressed the wound with oil. So Dr. Morton, of Glasgow, at a meeting of the Medico-Chirurgical Society of that city, said he had, some few years ago, made a comparative trial of several modes of surgical treatment, including the so-called antiseptic system. He had tried irrigation, carbolic acid putty, putty without carbolic acid, carbolic acid with oil, oil without carbolic acid, and a number of other medicaments. The result of this comparative trial—the only one, by the way, which he had yet heard of having been made—was to point, not to carbolic acid, but to oil, as being the most successful surgical application.

*Uterine Hemorrhage.*—Dr. Wenrick, in *Virchow's Archiv*, has an article on ergotin. He adds some therapeutical observations, made chiefly in treating cases of uterine hemorrhage with ergot. The following are his conclusions:—(1) The subcutaneous injection of ergot, especially in anæmic persons, stops uterine hemorrhage pretty quickly and permanently without any considerable unpleasant results; (2) The fact that a considerable portion of the fluid extract remains unabsorbed after subcutaneous injection renders the agent less trustworthy and the dose more difficult to regulate.

*Conium in the Treatment of Insanity.*—Dr. Daniel H. Kitchen (*Am. Jour. Insanity*, April, 1873), in an excellent article on this subject, speaks of the valuable experiments with *conia* hypodermically administered by Dr. J. W. Burman, of the West Riding Lunatic Asylum.

Twelve cases are related in which this drug was successfully given. His conclusions on its action are as follows: 1st. Muscular relaxation. 2d. Duration in proportion to dose. 3d. Physiological effect in proportion to purity of the article used. 4th. The brain is not affected directly by conium. 5th. Pulse and temperature both reduced after a full dose. 6th. A gentle perspiration covers the whole body as soon as the physiological effects are observed. 7th. No appreciable effect on any of the secretions. 8th. Quietness lasts from two to four hours, and then disappears, leaving only a sense of lessened muscular energy. 9th. Conium, not acting on the brain, may safely be given in all febrile diseases. 10th. Conium, when applied to the skin, causes slight redness.

Rusticus writes in the *Boston Medical and Surgical Journal* as follows: "Please say to the other country doctors, who don't know any more than we do, that pepsin can be very easily made an 'Aromatic Liquid Pepsin' by cutting up a calf's rennet-bag and bottling it up in a half gallon of pale sherry. It won't cost nearly so much; and mother used to feed her thirteen babies on it, at the rate of a teaspoonful to a cup of milk, with a little sugar mixed in, and a scratch of nutmeg on the top.

"I am told that you can buy rennet-bags cheap in Boston market. They are much better, I believe, after drying for weeks; and I should prefer them to pepsin. They will keep longer and better."

*Poisoning from Camphor.*—H. C. Hall, M.D., of Crawfordsville, Iowa (*The Clinic*, March 8, 1873), records a case of poisoning by spirits of camphor, which was taken by an old man, eighty years old, to relieve his diarrhœa. Ipecac, in large doses, combined with twenty grains of bromide of potassium, were given with success.

*Liquid Nourishment for Sick Stomach.*—An egg, well beaten up, to which add one pint of good milk, one pint of cold water, and salt to make it palatable; let it then be boiled, and when cold any quantity of it may be taken. If it turns into curds and whey it is useless.—*H. S. Halahan, in Dublin Medical Journal.*

Dr. T. Bates proposes (*The Clinic*) to substitute meal for bran in fracture-dressings as an absorbent. The meal, he claims, is equal to the other dressing, and is much superior in that it packs better and holds the limb immovable in the apparatus.

*Glycerine of Borax in Facial Erysipelas.*—Prof. D. M. Salazar, of the Hospital National, Madrid, reports that he has cured eight cases of facial erysipelas in forty-eight hours by this remedy. Notwithstanding the rapidity with which the affection disappeared, there were no consecutive pathological affections. In one case, the disease had existed three days before treatment was commenced, and there was bilious vomiting, intense cephalalgia, high fever, inflammation of the entire face, and some phlyctenulæ in the vicinity of the right lower eyelid and the root of the nose. He applied the solution to the diseased parts with a brush, and then covered them with a mask of raw cotton. After twenty-four hours, all the symptoms, local and general, were notably diminished, and the next day all the phlyctenulæ had disappeared and desquamation was commencing. All medication was then discontinued, except that a decoction of sambucus and althæa was used as a wash to favor the desquamation.—*El Amfit. Anat. Espan.*, Mar., 1873.

*Dysuria.*—Where there is difficulty in urinating, from stricture or other causes, and the sound does not readily pass, M. Cazenave, of Bordeaux, introduces a smooth piece of ice, about the size of a chestnut and of an oval form, into the rectum. The ice should be shoved past the sphincters and renewed from time to time. Almost always, after an hour or two, the urethral spasm abates, a certain quantity of urine is evacuated, and the bladder is emptied without extraordinary expulsive efforts on the part of the patient. In extreme cases it is also advisable to apply pounded ice externally, from the anus to the end of the penis. The same procedure is applicable to retention of urine caused by hypertrophy of the prostate; but in these cases the good effects are more slowly produced.—*La France Medicale.*

*Treatment of Cracked Nipple.*—Of this condition there are two varieties: The one is the result of violent efforts at suction; the epidermis is elevated, cup-shaped, and is fissured; to avoid it the mother should not give suck until the milk has accumulated in the breast. In the other variety, the milk deposits in the small clefts of the nipple in contact with the perspiration, and, decomposing, irritates and inflames the skin. The remedy consists in bathing the breast with lukewarm water, and afterwards sponging it with the following: tannin, one gramme; glycerine, ten grammes.—*Lyon Medicale.*

*Croup.*—Dr. W. W. Parker, of Richmond, Virginia (*Virginia Clinical Record*), relates a case of croup in which inhalations of lime proved efficacious. The most dense vapor is not at all unpleasant, and can be borne as well as the ordinary atmosphere of a heated room.

*Ergot in Abortion.*—Dr. Corson asks (*Medical and Surgical Reporter*, May 31st,) "would we dare ply a woman with ergot when pregnant and threatened with abortion? Certainly not." Now I claim and can establish by the testimony of at least fifty cases, that when threatened abortion is evidenced by pain and hemorrhage, ergot in *full doses* is the safest and most certain remedy known to the profession. Not for the purpose of checking hemorrhage and expelling the contents of the uterus, but to check hemorrhage and retain the fœtus *in situ* with safety to both mother and child.

*Capillary Puncture of the Pericardium by Subcutaneous Aspiration.*—In the *Lancet*, for March, 1873, we find a report, by Dr. Chairon, of a case of capillary puncture of the pericardium. The patient was a young soldier, who, at the end of an attack of pleurisy, presented all the symptoms of dropsy of the pericardium. M. Chairon introduced a capillary needle into the pericardial sac, and drew off a large quantity of sero-sanguinolent fluid, which quickly gelatinized. No accident attended the operation, and the following day he found the patient lounging about the passages of the hospital.

*Iodide of Potassium in Syphilitic Skin Diseases.*—Dr. McCall Anderson (*Med. News & Library*) lays down the following rules with regard to the employment of iodide of potassium in the treatment of syphilitic skin diseases:

1. The longer the interval which has elapsed between the contraction of the syphilitic taint and the development of the eruption, the more confidently may we substitute it for mercury.

*Sick Headache.*—Dr. H. McKay, of Tennessee (*Med. and Surg. Reporter*) says: For the benefit of Drs. Antie, Wilks and others, who suffer with headache, I ask you to publish the following formula, the efficacy of which I have tested time and again: ℞. Granulated muriate of ammonia, one teaspoonful; morphiæ acet, gr. j.; water, ℥ ss. Sig. Dose for an adult, two teaspoonfuls every ten minutes, precisely, till relief is obtained.

*Obstinate Vomiting of Pregnancy, cured by Enemata of Bromide of Potassium.*—Dr. Girabetti has successfully treated obstinate vomitings of pregnancy by enemata of bromide of potassium given in increasing doses; commencing with 6 grammes (about 92 grains) the first day, 8 grammes the second, and 10 grammes the third; after which the dose is lessened in proportion to the effect produced. In one case the vomitings were arrested by this treatment in three days.—*La Tribune Medicale.*

*Ascarides.*—Prof. Dame recommends the following treatment: One tablespoonful of common table-salt, dissolved in a pint of cold water, and enough of it used as an injection to fill up the rectum pretty well, not on kills the existing worms, but also destroys their larvæ. A salt-water injection every second or third night, until three our four had been used, has uniformly effectually relieved such cases as have come under my care.

*Remedy for Chronic Hoarseness.*—An eminent physician of Philadelphia contributes the following: In chronic hoarseness arising from thickening of the local chords and adjacent membrane the ammoniated tincture of guaiacum is often a very efficacious remedy. It may be appropriately mixed with equal parts of the syrup of senega, and a tea-spoonful of the mixture given two or three times a day.—*American Practitioner.*

*Cutaneous Anæsthesia* (*Centralblatt f. d. Med. Wissensch.*, March 22, 1873.)—Dr. Horvath, of Kiev, states that while water, ether, and mercury, at a temperature of —5° Centigrade, give intense pain before they produce cutaneous anæsthesia, alcohol and glycerin at the same temperature produce complete anæsthesia without any uncomfortable feelings.

*Sugar and Magnesia an Antidote to Arsenic.*—The *Mouvement Médical* relates various experiments conducted by Mr. Carl, with the result of showing that sugar mixed with magnesia may serve as an antidote in cases of poisoning by arsenious acid, in which cases, too, the internal use of the hydrated magnesia is most valuable.

*Diarrhœa Mixture.*—Dr. Wm. C. Lee, of Oregon has used the following recipe with great success in dysentery: ℞. Carbo. lingi.; creta prep., aa ℥ss; aqua cinnamond, ℥iv. Misce. Sig. Take one teaspoonful every two hours.

*For Dyspepsia.*—The following is with us a favorite prescription: ℞. Sub. nit. bismuth, ʒi; ext. nucis vomicæ, gr. iii. Make pills No. 12, and direct one pill morning, noon and night, one hour before eating.

*To Prevent Bed-Sores.*—Sir James Paget says the best wash for hardening the skin to prevent bed-sores, is one part of sweet spirits of nitre to three parts of water.

